# Three-day tracheal intubation manikin training for novice doctors using Macintosh laryngoscope, McGRATH MAC videolaryngoscope and Pentax AirwayScope

**DOI:** 10.1097/MD.0000000000023886

**Published:** 2021-01-29

**Authors:** Yuryo Murakami, Ryusuke Ueki, Miyuki Niki, Munetaka Hirose, Noriko Shimode

**Affiliations:** a Department of Anesthesiology and Pain Medicine, Hyogo College of Medicine; b Department of Anesthesiology, Fukuyama Medical Center; c Department of Anesthesiology, Amagasaki Chuo Hospital.

**Keywords:** Indirect laryngoscope, Macintosh laryngoscope, tracheal intubation, novice doctors, percentage of glottic opening score

## Abstract

**Background:**

We compared the intubation skills obtained by novice doctors following training using 3 instruments, the conventional Macintosh laryngoscope (Mac) and 2 types of indirect video-laryngoscopes (McGrath^TM^-MAC: McGrath (McG) and AirwayScope (AWS)), to determine the most appropriate instrument for novice doctors to acquire intubation skills, especially focusing on visual confirmation of vocal cords, during a 3-day intensive manikin training program.

**Methods:**

Fifteen novice doctors who did not have sufficient experience in endotracheal intubation (ETI) and consented to participate in this study were included. We used AirSim and AMT (Airway management Trainer) manikins. First, an experienced anesthesiologist instructed the trainees on using the 3 instruments for a few minutes. Then, after familiarizing themselves with each device for 10 minutes, the participants attempted ETI on the 2 manikins with the 3 devices used in random order. Intubations with each device were practiced and performed for 3 successive days. We assessed the percentage of glottic opening (POGO) score, successful intubation rate and tracheal intubation time for each participant, with each device, and on each day.

**Results:**

In the first manikin, AirSim, POGO scores in the McG and AWS groups were significantly higher than those in the Mac group on all 3 days (*P* < .0001). The number of intubation failures in the Mac group decreased from 2 cases on day 1, to 1 case on day 2 and zero cases on day 3. There were no failures in the McG and AWS groups on any of the days. With the second manikin, AMT, POGO scores in the Mac group were significantly lower than those in the McG and AWS groups on all 3 days. There were no intubation failures in the AWS group on all 3 days. In the Mac group, the number of intubation failures decreased from 3 on day 1, to 2 on day 2 and zero failures on day 3. In the McG group, there were only 3 failures on day 1.

**Conclusion:**

The 2 types of indirect video-laryngoscopes (McGRATH and AirwayScope) were demonstrated to be suitable instruments for novice doctors to achieve higher POGO scores in a 3-day intensive ETI training.

## 1 Introduction

Tracheal intubation is 1 of the core skills to be learned by novice doctors during their training period. However, tracheal intubation with use of the Macintosh laryngoscope requires some amount of practice.^[1–5]^ Several indirect video-laryngoscopes have been developed and their efficacy for tracheal intubation has been amply reported.^[6–11]^ However, there are only a few reports on repeated intubation practice over a short time period, such as 3 days, for novice practitioners.^[3,4,9]^ Among these 3 previous reports, laryngeal view, which was assessed based on the Cormack & Lehane classification, was the focus of only 1 of the studies. However, there is no report comparing vocal cord visualization by inexperienced doctors, assessed using the percentage of glottic opening (POGO) score, with the use of the Macintosh laryngoscope versus indirect video-laryngoscopes during intensive short-term training. Hence, we aimed to evaluate the efficacy of indirect video-laryngoscopes in the training of unskilled novice doctors.

**Figure 1:**
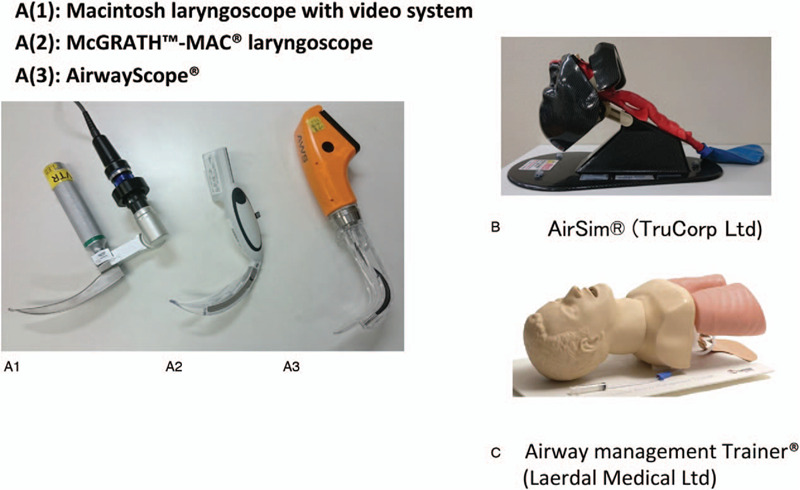
Laryngoscopes and manikins used in this study. A(1): Macintosh laryngoscope with video system, A(2): McGrath-Mac laryngoscope, A(3): AirwayScope. B. AirSim (TruCorp Ltd.) manikin, C. Airway management Trainer (Laerdal Medical Ltd.).

In this study, we evaluated which of the 3 instruments, the conventional Macintosh laryngoscope and 2 types of indirect video-laryngoscopes (McGRATH and AirwayScope), is appropriate for the training of novice doctors, facilitating their quick acquisition of the skills and techniques required for tracheal intubation over 3 days of intensive training (Fig. 1A), with a focus on POGO scores in relation to the difficulty of tracheal intubation.^[12]^

## 2 Methods

### 2.1 Study design and settings

This study protocol was approved by the Institutional Review Board of Hyogo College of Medicine (approval number 1527). The subjects were novice doctors, with experience of less than 5 intubations, who agreed to participate in this study. Written informed consent was obtained from each participant. Two types of manikins, AirSim (TruCorp Ltd., United Kingdom) and AMT (Airway management Trainer: Laerdal Medical Ltd., Norway), were used in this study (Fig. 1B, 1C). The laryngoscopes used were the Macintosh laryngoscope with video system (Mac, Macintosh laryngoscope, WelchAllyn. Inc., USA, and Videosystem; Machida Ltd.: Chiba, Japan), McGRATH-MAC laryngoscope (McG, Covidien Japan Inc., Tokyo, Japan or Medtronic Inc., Dublin, Ireland) and AirwayScope (AWS, Nihon Kohden Co., Tokyo, Japan). The Macintosh laryngoscope with video system allows the evaluator to observe the field of view during the trial. Therefore, with this device, the novice doctors identified the vocal cords by directly observing the airway under direct laryngoscopy, and the evaluator judged the POGO score and success of tracheal intubation on the screen of the video system. A size 7.5-mm cuffed tracheal tube (Covidien Japan Inc., Tokyo, Japan), MAC 4 laryngoscope blade for McG (Covidien Japan Inc., Tokyo, Japan) and standard type intlock blade for AWS (Nihon Kohden Co., Tokyo, Japan) were prepared.

### 2.2 Training

During the training, an anesthesiology specialist introduced the 3 devices to the subjects, instructing them regarding handling the instruments and the practical methods of tracheal intubation. Then, the participants practiced tracheal intubation on the AirSim manikin using the 3 devices for 10 minutes each (total 30 minutes).

### 2.3 Outcomes

The primary outcome was the best POGO score among the 3 instruments. The secondary outcome was time to successful ETI over the 3 days.

### 2.4 Measurements

After the practice, the participants were expected to perform intubations with the 3 scopes in both of the manikins, the order of use of each device in each manikin being decided by a randomized lottery. The intubation procedures were performed on the AirSim and AMT in turn, and were evaluated by the anesthesiology specialist. The number of attempts allowed for each trial was not limited. The parameters assessed were POGO score (example, 0%: invisible, 50%: half visible, 100%: entire vocal cords visible) and intubation time (total, time from holding the device till visualization of the vocal cords, and time from visualization of the cords to passage of the tube through the vocal cords). If the time to performing tracheal intubation exceeded 180 seconds, the attempt was considered a failure and was discontinued. We evaluated the success rates of each trial and the results over the 3 days.

### 2.5 Statistical analysis

In this study, sample size was calculated based on a preliminary study that evaluated the POGO score required for 6 participants to identify the vocal cords. The minimum and second lowest mean (SD: standard deviation) POGO (%) scores with the AirSim manikin were 51% (25.8) and 83% (6.1), respectively. To detect a difference in POGO score with a power of 0.8 and level of significance of p=0.05, we estimated that 11 operators would be adequate for each device.

Statistical analysis was performed using 1-way repeated measures analysis of variance with post hoc Tukey's multiple comparison test and 2-way repeated measures analysis of variance using Graph Pad Prism software. *P* < .05 was considered a significant difference. The Macintosh group was used as a control.

## 3 Results

In total, 15 trainee doctors performed intubations with each of the laryngoscopes on each of the manikins every day for 3 successive days.

### 3.1 Intubation of AirSim

**Figure 2:**
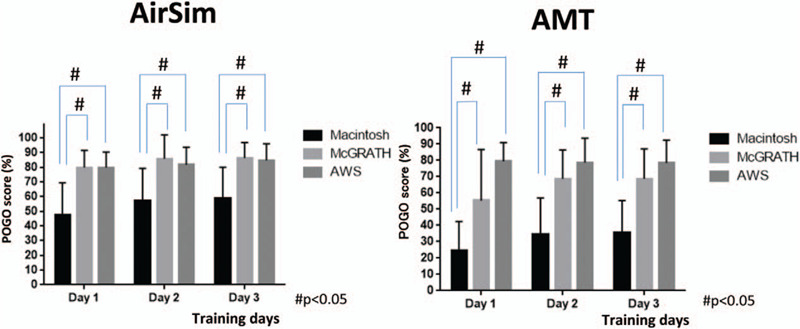
POGO scores in each group over the 3 training days. (AirSim vs. AMT). #POGO: percentage of glottic opening. ∗In both AirSim and AMT, POGO scores with the McG and AWS were higher than those with the Mac on all 3 days.

In the first manikin, the AirSim, POGO scores in each group were as follows. The values [mean (SD)] were 48% (21.0) on day 1, 57% (11.3) on day 2, and 59% (10.1) on day 3 in the Mac group. On the other hand, POGO scores in the McG group were 80% (11.3) on day 1, 86% (15.8) on day 2, and 86% (20.6) on day 3, and those in the AWS group were 80% (10.4) on day 1, 82% (11.5) on day 2, and 85% (10.8) on day 3. POGO scores in the McG and AWS groups were significantly higher than those in the Mac group on all 3 days (p value and 95% confidence interval, McG vs. Mac: *P* < .0001, 22.45 vs 36.22, AWS vs Mac: *P* < .0001, 20.56 to 34.33) (Fig. 2). The number of intubation failures in the Mac group decreased progressively from 2 failures on day 1, to 1 event on day 2 and zero failures on day 3. There were no failures in the McG and AWS groups throughout the 3-day training period.

**Figure 3:**
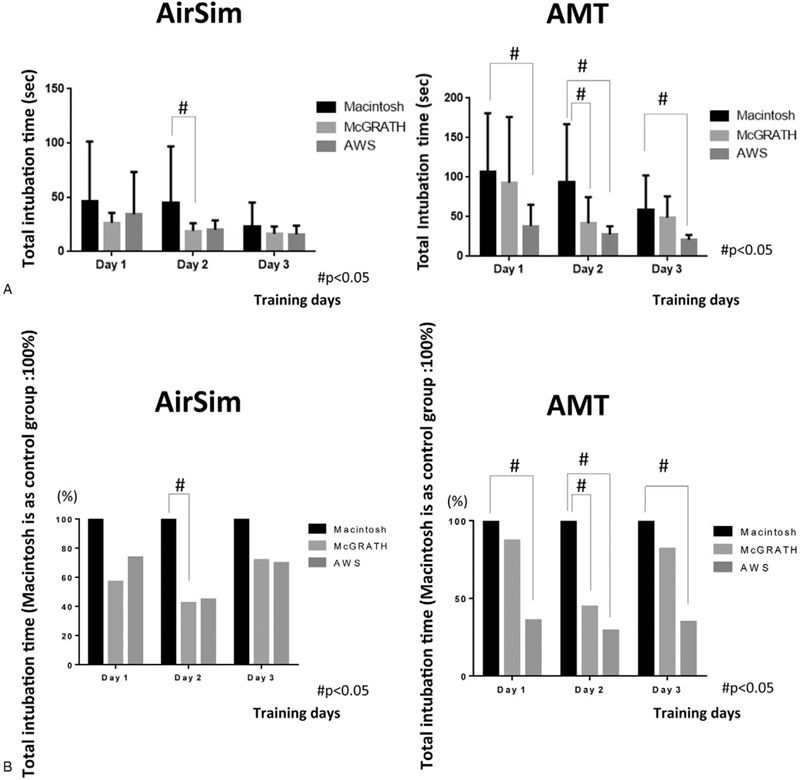
Total intubation time in each group over the 3 days (AirSim vs AMT). Actual time data was shown in Figure 3(A). Figure 3(B) indicates the percentage change relative to the Macintosh group (considered as 100%). # In the AirSim study, compared with the Mac group, total intubation time was significantly shorter in the McG group on day 2. # In intubation training with the AMT manikin, the total time to tracheal intubation with the AWS was significantly shorter than that with the Mac on all 3 days. Further, the time to intubation with the McG was significantly shorter than that with the Mac on day 2.

**Figure 4:**
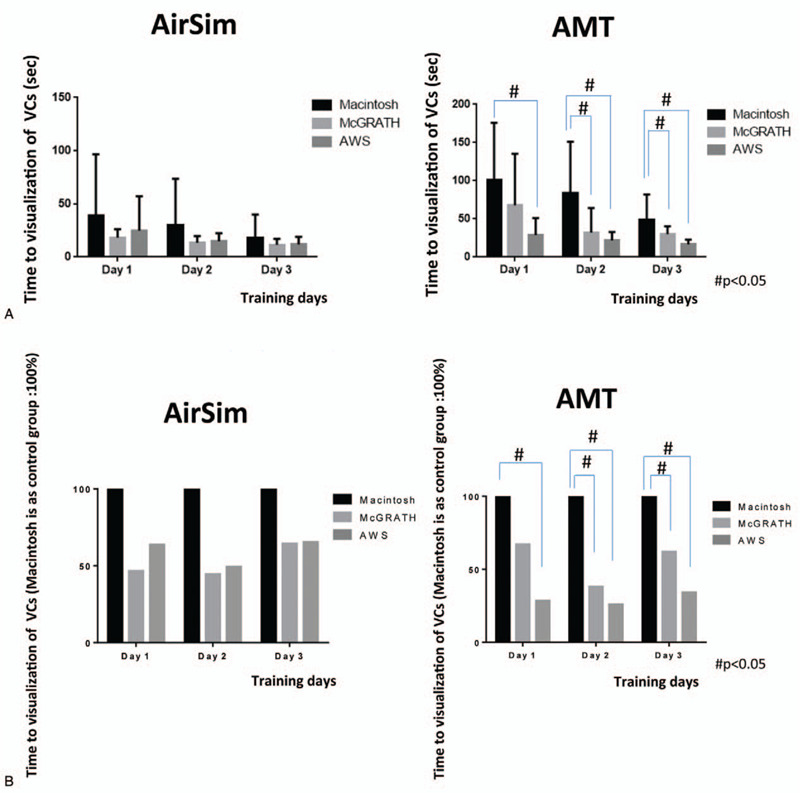
Time between holding the laryngoscope and vocal cord (VC) visualization in each group on the 3 days (AirSim vs AMT). Actual time data was shown in Figure 4(A). Figure 4(B) indicates the percentage change relative to the Macintosh group (considered as 100%). #In the AMT study, the time until visualization of the VCs was significantly shorter with the McG than the Mac on days 2 and 3, and that with the AWS was significantly shorter than with the Mac on all 3 days.

**Figure 5:**
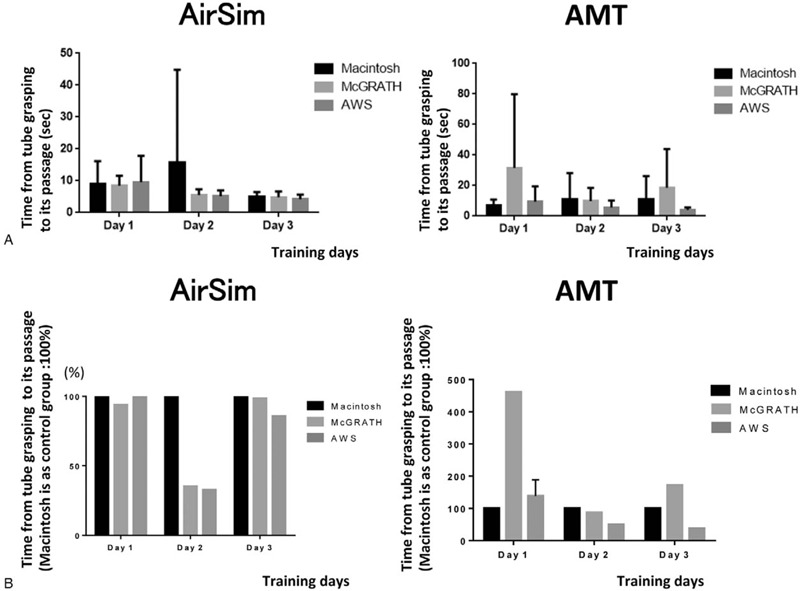
Time between grasping the tracheal tube and tube passage through the vocal cords in each group over the 3 days (AirSim vs AMT). Actual time data was shown in Figure 5(A). Figure 5(B) indicates the percentage change relative to the Macintosh group (considered as 100%). ∗There were no significant differences in the AirSim study and the AMT study.

Since we defined the Macintosh group as the control group, we compared intubation attempts with the McG and AWS by each participant with those with the Mac, and created images to show these comparisons. Figures 3(A), 4(A) and 5(A) show actual individual time data for each trial, while Figures 3(B), 4(B) and 5(B) indicate the values after conversion and expression as the percentage change relative to the Macintosh group (representing 100%). Compared with the Mac group, total intubation time was significantly shorter in the McG group on day 2 (Fig. 3 (A, B)).

### 3.2 Intubation of AMT

In the second manikin, AMT, POGO scores in each group were as follows. The values [mean (SD)] were 25% (17.1) on day 1, 35% (21.6) on day 2, and 35% (19.2) on day 3 in the Mac group. On the other hand, those in the McG group were 55% (30.3) on day 1, 69% (17.1) on day 2, and 69% (17.7) on day 3, while those in the AWS group were 79% (11.2) on day 1, 79% (14.5) on day 2, and 78% (13.6) on day 3 (Fig. 2). POGO scores were highest in the AWS group, with high average scores of the 3 days, which indicates sufficient visualization of the vocal cord from day 1. POGO scores in the McG group increased from an average of 55% on day 1 to an average of 69% on day 3. On the other hand, POGO scores in the Mac group increased from 26% on day 1 to 35% on day 3. However, average POGO scores over the 3 days were significantly higher in the McG and AWS groups as compared to those in the Mac group (*P* value and 95% confidence interval, McG vs. Mac: *P* < .0001, 25.27 vs 40.06, AWS vs Mac: *P* < .0001, 39.83 vs 54.62).

There were no intubation failures in the AWS group over the entire training period. In the Mac group, the number of intubation failures decreased from 3 on day 1, to 2 on day 2 and zero failures on day 3. In the McG group, there were 3 intubation failures only on day 1. Compared with the Mac group, total intubation time and the time from holding a device to visualizing the vocal cords were significantly shorter in the AWS group on all 3 days (Fig. 3 (A, B), 4 (A, B)). In addition, similar significant differences in total intubation time and time from holding a device to visualization of the vocal cords were recognized for the McG group on day 2 (Fig. 3) and days 2 and 3 (Fig. 4), respectively. There were no significant differences in the time from visualizing the vocal cords to tracheal intubation between the 3 scopes (Fig. 5(A, B)).

## 4 Discussion

The results of this study indicate that the AWS is preferable to the other laryngoscopes evaluated in this study for initial training of novice doctors, to facilitate their acquisition of the skills required for tracheal intubation. McG and AWS seemed to be superior to the Macintosh laryngoscope in terms of the achievement of excellent POGO scores.

Recently, the superiority of indirect video-laryngoscopes has been recognized. Therefore, the use of these devices is likely to increase in the future. As previously reported, indirect video-assisted laryngoscopes are associated with a higher success rate of tracheal intubation by inexperienced practitioners.^[9–11]^ In addition, indirect video-laryngoscopes permit sharing of the visual field via a screen, allowing visualization of the vocal cords by both the novice practitioner and the instructor, thus facilitating intubation and preventing vocal cord injury, including mucosal injury, laryngeal edema, hematoma, hoarseness and arytenoid cartilage subluxation. However, there are only a few reports on intubation training for novice practitioners performed over a short time period.^[3,4,9]^ On the other hand, a Macintosh laryngoscope is still preferable during training for the management of cases of emergency oral bleeding and for removal of foreign bodies.^[13,14]^ Besides, sufficient attention to prevention of dental injury during tracheal intubation is necessary.^[15]^ Hence, this study might serve as a reference for tracheal intubation training of novice doctors.

A point of interest should be noted in Figure 5. A long error bar was noticed in the Macintosh group on day 2. This phenomenon was caused by the very long time (114 seconds) taken for tracheal intubation by 1 of the participants, who could not handle the tracheal tube very well. This might also occur with impatience on the practitioner's part, leading to them hurrying with the process, resulting in many mistakes. This result might also indicate unstable and immature intubation skills.

Our study has some limitations. Since this was a manikin study, the results cannot be extrapolated to patients who have loose teeth and restricted mouth opening. In addition, indirect video-laryngoscopes have disadvantages of poor visibility in the presence of vomitus, oral secretions and bleeding in the mouth, and cloudiness due to exhalation.^[13,14]^ Therefore, further investigations that take into account various situations and patient factors that cause difficult airway are necessary.^[16–18]^

In summary, our study indicates that the 2 types of indirect video-laryngoscopes, AirwayScope and McGRATH, are appropriate instruments for the initial training of novice doctors via short-term instruction, to enable quick acquisition of the skills required for successful tracheal intubation, as indicated by the high POGO scores with the 2 types of indirect video-laryngoscopes compared with the Macintosh laryngoscope. Our study also reveals that intensive training by repeated performance of intubation procedures over a 3-day training period facilitates skill improvement and enhances acquisition of the skills required for endotracheal intubation.

## 5 Conclusions

This study showed that the 2 types of indirect video-laryngoscopes evaluated (McGRATH and AirwayScope) might be superior to the Macintosh laryngoscope for training novice doctors about tracheal intubation, as seen by the attainment of excellent POGO scores with the use of these devices in a 3-day intensive tracheal intubation training program on manikins.

## Acknowledgment

We would like to thank all the novice doctors who participated in this study.

## Author contributions

**Conceptualization**: Noriko Shimode

**Data curation**: Noriko Shimode

**Formal analysis**: Yuryo Murakami, Ryusuke Ueki, Noriko Shimode

**Investigation**: Yuryo Murakami, Miyuki Niki, Noriko Shimode

**Methodology**: Miyuki Niki, Noriko Shimode

**Project administration**: Noriko Shimode

**Resources:** Noriko Shimode

**Software**: Ryusuke Ueki, Noriko Shimode

**Supervision**: Munetaka Hirose, Noriko Shimode

**Validation**: Yuryo Murakami, Ryusuke Ueki, Miyuki Niki, Munetaka Hirose, Noriko Shimode

**Visualization**: Yuryo Murakami, Ryusuke Ueki, Munetaka Hirose, Noriko Shimode

**Writing – original draft**: Yuryo Murakami, Ryusuke Ueki, Miyuki Niki, Munetaka Hirose, Noriko Shimode

**Writing – review and editing**: Yuryo Murakami, Ryusuke Ueki, Miyuki Niki, Munetaka Hirose, Noriko Shimode

## Abbreviations

Abbreviations: AMT = airway management trainer, AWS = airwayscope, ETI = endotracheal intubation, Mac = Macintosh laryngoscope with video system, McG = McGRATH-MAC laryngoscope, POGO = percentage of glottic opening.
